# Role of the Endothelium during Tumor Cell Metastasis: Is the Endothelium a Barrier or a Promoter for Cell Invasion and Metastasis?

**DOI:** 10.1155/2008/183516

**Published:** 2009-03-05

**Authors:** Claudia Tanja Mierke

**Affiliations:** Biophysics Group, Center for Medical Physics and Technology, Friedrich-Alexander-University Erlangen-Nuremberg, 91052 Erlangen, Germany

## Abstract

The malignancy of cancer disease depends on the ability of the primary tumor to metastasize to distant organs. The process of the metastasis formation has largely been analyzed, but still main pathways regarding the extravasation step at the end of the metastasis formation process are controversially discussed. An agreement has been reached about the importance of the endothelium to promote metastasis formation either by enhancing the growth of the primary tumor or by homing (targeting) the tumor cells to blood or lymph vessels. The mechanical properties of the invading tumor cells become the focus of several studies, but the endothelial cell mechanical properties are still elusive. This paper describes the different roles of the endothelium in the process of metastasis formation and focuses on a novel role of the endothelium in promoting tumor cell invasion. It discusses how novel biophysical tools and in vivo animal models help to determine the role of the endothelium in the process of tumor cell invasion. Evidence is provided that cell mechanical properties, for example, contractile force generation of tumor cells, are involved in the process of tumor cell invasion.

## 1. Introduction

**Figure 1:**
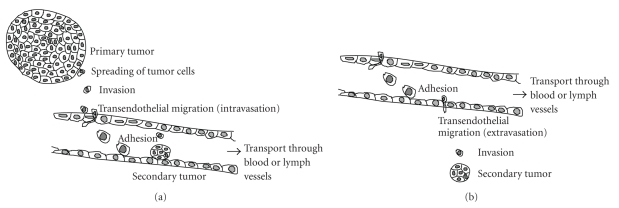
Metastasis formation involves several steps. Two possible ways of metastasis formation: (a) first, single tumor cells disseminate form the primary tumor and invade the extracellular matrix, intravasate into blood or lymph vessels, and get transported and adhere to the endothelium. Secondly, (a) invasive tumor cells grow in vessels and do not transmigrate or (b) invasive tumor cells transmigrate through the endothelium, invade and from a secondary tumor in the targeted tissue.

The coordinated regulation of cell adhesion and cell motility especially the migration of cells in a three-dimensional (3D) environment (cell invasion) is essential for the development and function of almost all multicellular organisms. The transformation of cell-cell and cell-matrix adhesions as well as the loss of contact responsiveness is an indicator for the malignancy of tumors [1, 2]. Cell invasion is a driving force in malignant tumor diseases, for example, in the progression of primary tumor outgrowth and in the process of metastasis formation. The malignancy of neoplasms is determined by the capability of single tumor cells to invade into the surrounding tissue, intravasate into blood or lymph vessels, be transported, possibly extravasate and invade into the connective tissue, proliferate, and finally metastasize [3, 4] (Figure 1).

**Figure 2:**
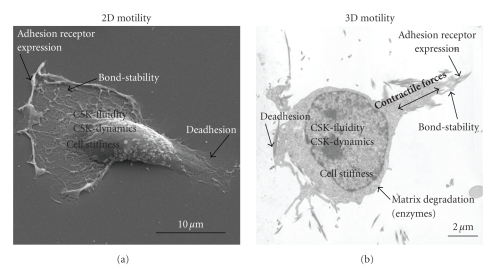
Prerequisites for tumor cell migration on a 2D substrate and tumor cell invasion in a 3D extracellular matrix. (a) Scanning electron microscopic image of an MDA-MB-231 breast carcinoma cell on a planar glass and (b) transmission electron microscopic image of an MDA-MB-231 cell invaded in 3D collagen gel matrix. (a) For proper 2D motility, a cell needs to adhere/deadhere fast, remodel its cytoskeleton fast, and possess a high CSK-fluidity. (d) For proper 3D motility, a cell needs, in addition to the 2D motility properties, to generate high contractile forces, and the cells needs to overcome the viscous drag of the surrounding extracellular matrix to move forward.

Cell invasion is a central step in the process of metastasis formation. The prerequisites for cell invasion involved in the metastasis formation process are (1) cell adhesion (adhesion strength), (2) force generation, (3) detachment (deadhesion and cytoskeletal remodeling), and (4) matrix composition and remodeling via enzymes (Figure 2). The balance of all these parameters determines the cell invasiveness, for example, the migration speed in a 3D extracellular matrix. All these considerations might play a more or less important role in cell invasion depending on the cell type and tissue origin, gene, or protein expression pattern of a cell, the environment (growth factors, matrix constituents, and mechanical stiffness), and adjacent cells. In line with cell invasion, the interaction of a cell with the neighboring cells underlies also mechanical properties of the cytoskeleton of the two adjacent cells.

Alterations of cell-cell and cell-matrix interactions are mirrored in the cytoskeleton and account for the ability of tumor cells to cross tissue boundaries and to disseminate to distant organ sites. Many adhesion molecules implicated in tumor metastasis have been identified, but there exist only a few studies with a broad designed approach. Several adhesion or cell surface receptors have been identified to act either as negative, for example, E-Cadherin [5] or positive factors, for example, *α*v*β*3, CD24, and CXCR2 of tumor invasion and metastasis formation [6–8]. The focus of this paper is on the processes of cell invasion and transendothelial migration of tumor cells as well as on their prerequisites including the presentation of novel biophysical tools and in vivo metastasis models. The emphasis is on the function of the endothelium in the process of metastasis formation, such that it promotes tumor cell invasion.

## 2. Tumor Cell Invasion

### 2.1. How Do Cell-Matrix Adhesion Receptors Influence Tumor Cell Invasion?

To generate tractions especially in a 3D extracellular matrix, the cells need to have a tight connection between the extracellular matrix and the intracellular cytoskeleton via focal adhesions and cell-matrix receptors. This connection is commonly facilitated by transmembrane proteins like integrin cell adhesion receptors. Integrins are heterodimeric proteins that are involved in cell adhesion, migration, and invasion processes and regulate the cell cycle. The protein family of human integrins comprises 18 *α*- and 8 *β*-subunits [9]. At least 24 different *α*-*β*-heterodimer combinations have been identified, that are capable of binding to a large variety of ligands, among them were components of the extracellular matrix (e.g., collagen, fibronectin, and laminin) as well as binding partners on adjacent cells (e.g., VCAM-1 and PECAM-1) [10–13]. The physical link between these adhesion receptors and the actin cytoskeleton via intracellular focal adhesion proteins mediates the bidirectional force transmission and the biochemical signal transduction across the plasma membrane [14, 15]. The major signaling pathways activated by integrins through their *α*-subunits are different. The activation of *α*4 and *α*9 integrin subunits leads to decreased cell spreading and increased cell migration on a planar substrate [16–18]. This is in contrast to the activation of other integrin *α*-subunits (e.g., *α*1, *α*5, *α*v, *α*3, *α*6, and *α*IIb) that lead to enhanced cell spreading and decreased cell migration [19–23] and show different bindings to fibronectin via the canonical integrin recognition site arginine-glycine-aspartic acid (RGD) [24–28]. Furthermore, the *α*5*β*1 integrin regulates the *α*v*β*3-mediated adhesions and migration on the extracellular matrix [29]. The formation, activation, and regulation of focal adhesion proteins in the focal adhesions and their physiological function are pivotal and have been the focus of intense research efforts and may have opposite effects depending on the integrin subunit type [9], but the mechano-regulating function, for example, in the process of cell invasion is still elusive. The *α*v*β*3, *α*v*β*6, and *α*6*β*4 integrins have been described to act as enhancers of tumor cell invasion and metastasis formation [30–32]. Their role in the process of metastasis formation and their impact on transendothelial migration of tumor cells are still unclear.

The *β*1 integrins have also been reported to promote tumor cell migration in a matrigel [33]. To make it more confusing, the role of the *α*5*β*1 in the process of 3D cell invasion, tumor growth and metastasis formation is controversially discussed because several reports described an invasion enhancing, tumor growth promoting, and metastasis increasing role that is inversely correlated with E-cadherin expression [34, 35], but others reported it vice versa [36–38]. The differences of the results might be explained by the use of different tumor cell lines. The correlation of the expression of one gene alone might not be helpful in determining the malignant potential of a tumor. The combination of gene expression and biophysical measurements of the cell's mechanical properties could be more sufficient because the expression a certain integrin by itself changes the cytoskeletal structure of a cell [39]. Combined with gene expression data, the overall cellular stiffness or the cytoskeletal dynamics of a cell could explain cellular behavior, for example, cell invasion and help to understand the general mechanisms important for the regulation of this process [6].

### 2.2. Role of the Extracellular Matrix Composition and Mechanical Properties of the Tissue

The mechanical properties of the extracellular matrix determine the gene expression patterns, the differentiation, proliferation, and cell mechanical properties [40–42]. The ability to generate forces might also be influenced by the mechanical properties of the 3D environment of the cell [43]. Furthermore, the environmental properties of the primary tumor might also determine the secondary tumor formation site maybe because of similar mechanical properties of the primary tumor tissue and the new metastasis formation site. Adhesion strength and cell stiffness determine the strength of the contractile forces [43] and might influence the ability of cells to invade into the extracellular matrix and to transmigrate through an endothelial cell monolayer and the basement membrane surrounding the vessels. The ability to remodel their cytoskeleton to squeeze through the pores of the extracellular matrix (amoeboid migration) with or without the generation of contractile forces is important for the invasion speed, invasion depth, and finally the ability to form metastasis. For the migration in a 3D matrix, the CSK-fluidity may play also an important role, the higher the CSK-fluidity the higher the invasiveness of the cells (Figure 2).

### 2.3. Enzymatic Modulation of the Mechanical Properties of a 3D Connective Tissue

Changes of the extracellular matrix composition and structure (e.g., matrix pore size) influence the mechanical parameters of the matrix that could impact cell invasiveness via changing the mechanical properties inside the cell. These changes of the extracellular matrix's mechanical properties could origin from the invading cell itself. For example, cell shape changes such as protrusion dynamics of mesenchymal tumor cells were monitored in combination with matrix deformation due to enzymatic matrix remodeling using a 3D collagen matrix embedded with polystyrene beads [44]. The cells themselves produce enzymes either membrane-bound (e.g., MT-MMPs) or secreted to modulate the adjacent extracellular matrix [33]. The expression of collagenases MMP-14, MMP-12, and MMP-9 increase the invasiveness of tumor cells migrating through a collagen fiber network or through a Boyden-chamber (transwell membrane) [33]. MMP-9 cooperates with the *α*v*β*3 integrin in supporting breast carcinoma cell migration using transwell membrane assay [20]. The inhibition of these enzymes using a cocktail of inhibitors leads to reduced or impaired invasion of tumor cells and changes the prior mesenchymal invasion to an amoeboid invasion type [33]. The production of sheddases leads to the cleavage (e.g., ADAMs = a disintegrin and metalloprotease domain) of several adhesion receptors like *α*6 integrin and *α*v*β*3 integrin, reduces cell adhesion, and increases motility [22, 45, 46]. ADAMs are a family of type I transmembrane glycoproteins that have the ability to bind to integrins and disrupt their adhesion to the extracellular matrix [47–49]. The ADAM-15 sheddase contains an RGD-peptide motif and can hence act as a natural binding partner for *α*v*β*3 integrin and reduce cell adhesion, increasing migration and invasion of tumor cells [50]. Despite the binding to *α*v*β*3 integrin, the ADAM-15 interacts also with the *α*5*β*1 integrin [51]. Hence, the expression of sheddases like ADAM-15 is associated with a poor diagnosis of the cancer disease [52] and supports the interaction of prostate cancer cells and endothelial cells in the process of metastasis formation [53]. Confirming that the overexpression of MMP-14 increased the invasiveness of the cells, more cells were able to invade and they invade deeper into the gel via enhanced motility (or a higher adhesion site turn over rate). The cutting of the extracellular part of membrane-bound receptors reduced their cell surface expression that leads to decreased adhesion strength and decreased traction generation. All these enzymes alter either the extracellular matrix adjacent to the invading cell or the adhesion receptor expression on the migrating cell itself. This might influence, in turn, the adhesion strength (e.g., the adhesion process) and the generation of contractile forces (Figure 2).

## 3. Interactions of Tumor Cells and Endothelial Cells

### 3.1. Transendothelial Migration: Intravasation and Extravasation of Tumor Cells

The metastasis formation process begins with the dissociation of single or clustered tumor cells from the primary tumor and is followed by extracellular matrix invasion, entrance into blood or lymph vessels (intravasation), and transport to other tissue sites of the body (Figure 1). It has been reported that tumor cells sometimes escape from the microvasculature (extravasation), invade into the tumor-type dependent specified target tissue, and form a secondary tumor in distant organs [3, 54, 55]. The mechanisms of the tumor-type specific targeting of secondary tumor formation are almost unknown. Some people speculate about mechanical properties of the tissues determining different target-organ selections for secondary tumor formation [56, 57]. The extravasation process as well as the intravasation process involves adhesion of tumor cells to endothelial cells and the transmigration through the endothelium and underlying basement membrane [3, 58–60]. For several tumor cell types, it has been reported that they are able to pass the endothelial barrier (perivascular metastasis) [60–65]. Nevertheless, the extravasation step is not the only mechanism for metastasis formation, it is also possible that tumor cells intravasate into vessels, but they never extravasate. These tumor cells grow inside the vessels on the endothelial cell layer and form the metastasis at these sites (intravascular metastasis) [4]. Despite the extravasation step performed, the function of the endothelial monolayer in the metastasis formation process is thought to be crucial in that it can actively regulate metastasis formation by either allowing or blocking the adhesion, and possibly transmigration, of tumor cells and thereby determining the target organ of secondary tumor formation [62–64]. The endothelial cell function in the process of metastasis formation is still unclear, and the effect of the endothelium even to promote tumor cell invasion has recently been reported, but the mechanism is poorly understood [6]. More insights might bring novel in vivo models on metastasis formation.

### 3.2. In Vivo Models: Vascular and Lymphatic Routes of Metastasis Formation

Metastasis formation occurs via the vascular system as well via the lymphatic system [4, 66–68]. Tumor cells can form intravascular and perivascular metastases [3, 4, 69] (Figure 1). Any type of cancer process, including tumor cell motility and invasion, as well as metastasis formation can be measured using animal models in combination with fluorescent proteins that stain the injected tumor cells as well as the host tissue. Naturally fluorescent proteins, especially those with long excitation wavelengths, visualize in real time, aspects of tumor initiation, tumor growth, and metastasis formation in living animals [66, 68, 70–73]. The benefit of these novel in vivo rodent metastasis models allows us to study migration directly in live blood or lymph vessels and tumor-host interactions in the presence or in the absence of anticancer drugs or chemotherapy drugs [67, 71, 74, 75]. Besides, the injection of tumor cells whole tissue explants (named orthotropic model) can be transferred into nude mice and studied for response to drugs or vascularization [71, 76]. These studies show that tumor cells metastasize via vascular and lympathic vessels [4, 66–68]. The lymphatic route (lymphatic system) of the tumor metastasis formation is less well understood compared to the vascular route (circulatory system) [77]. Using the real-time imaging of fluorescently labeled tumor cells that were injected in the inguinal lymph node, tumor cell survival and migration to the axillary lymph node were measured. In addition, the effect of pressure on tumor cell shedding into lymph vessels and migration to the lymph node was analyzed. Due to the clinical studies indicating that high interstitial fluid pressure in the tumor is correlated with a poor diagnosis [78–80], this lymphatic animal model shows that increased pressure on the tumor increased the numbers of shedded tumor cells that may lead to higher lymphatic metastasis formation [68]. The mechanical properties of the extracellular matrix (in this case of the tumor) change the cellular properties toward higher motility and invasiveness. A combination of these in vivo models with biophysical measurements will help to get more insights and reveal the mechanisms why some tumors metastasize and others do not and what role the endothelium plays.

### 3.3. Does the Endothelium Represent a Barrier for Tumor Cell Invasion?

**Figure 3:**
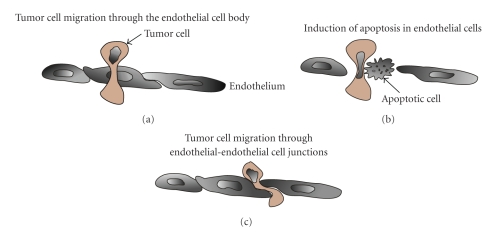
Three currently discussed strategies for tumor cell transmigration through an endothelial cell monolayer. (a) The tumor cell transmigrates through the cytoplasm of the cell body. (b) The tumor cell induced apoptosis in the adjacent endothelial cell and migrated through the hole in the endothelial cell layer. (c) The tumor cell transmigrates through the endothelial cell-cell contacts without permanent destroying the endothelial cell monolayer.

It is still unclear whether tumor cells transmigrate through the cytoplasm of endothelial cells similar to leukocytes, or destroy endothelial cells [81], or transmigrate between two adjacent endothelial cells by Src-mediated disruption of their cell-cell connections (VE-cadherin-*β*-catenin) [61]. It has been reported that transmigrating tumor cells are able to overcome the endothelial barrier by inducing changes within the endothelial cell monolayer, including the upregulation of adhesion molecule receptor expression [82], the reorganization of the cytoskeleton [83], Src-mediated disruption of endothelial cell-cell-adhesions [61], the formation of “holes” within the endothelial layer [84], and the induction of apoptosis [81] (Figure 3). Furthermore, tumor cell invasion may use similar invasion strategies as leukocytes, for which the endothelium acts as a barrier and greatly reduces invasion rates [85].

### 3.4. How Do Cell-Cell Adhesion Receptors Influence Tumor Cell Invasion?

**Figure 4:**
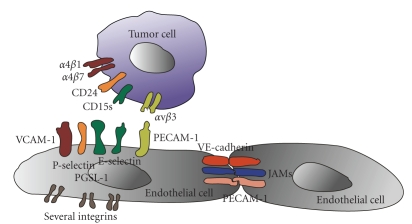
Interactions between tumor and endothelial cells during tumor cell adhesion and transmigration.

Beside cell-matrix receptors, cell-cell adhesion receptors play a role in cell invasion and especially facilitate transendothelial migration of tumor cells as well as leukocytes. It has been reported that *α*v*β*3 integrin as well as glycosylphatidylinositol-anchored CD24 protein facilitates tumor cell transmigration and enhances tumor cell invasion in a 3D collagen fiber network [6, 8, 86] (Figure 4). Endothelial cells expressed L1-CAM and PECAM-1 have been described as counter-receptors that interact with cancer cell *α*v*β*3 integrin [87, 88]. Endothelial cells expressed L1-CAM, and P-selectin have been described as a ligands for CD24 [7, 89]. A loss of E-cadherin cell-cell contacts is important for single cell invasion and has recently been discovered as a tumor suppressor gene [5]. The *α*4*β*1 integrin interacts either with VCAM-1 or CD14 to enhance tumor cell migration or invasion [11, 90]. It has to be ruled out how the interaction of tumor cells and endothelial cells through these receptors might, in turn, alter the mechanical properties of both cell types and change their invasive potential.

### 3.5. Role of Cytokines, Chemokines, or Other Stimuli in Tumor Cell Invasion and Transmigration

The scatter factor/hepatocyte growth factor (HGF) has largely been analyzed to enhance tumor cell migration on 2D substrates in in vitro cell culture assays [91]. Consistent to in vitro experiments, in vivo experiments have shown that this factor increased tumor cell spreading, dissemination, and metastasis formation in mice [91]. Recently, it has been shown that HGF influences the expression of integrin receptors, which in turn mediates the secretion of MMPs [92]. Despite this interaction, HGF ligand binding to the Met receptor as well as the *α*6*β*4 integrin can independently enhance tumor cell invasion [32].

**Figure 5:**
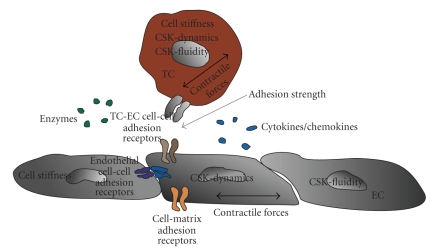
Prerequisites for tumor cell transendothelial migration. Single tumor cells adhere to the endothelium via cell adhesion receptors like integrins, muccins, or immunoglobulins and their counter-receptors on endothelial cells. Many other factors like enzymes, cytokines/chemokines influence these interaction as well as biomechanical properties of both cells (CSK-dynamics, CSK-fluidity, cell stiffness, and contractile force generation).

Cytokines affect the barrier function of an endothelial cell monolayer (Figure 5). For example, the function of the endothelial cell barrier against both leukocyte trafficking and tumor cell transmigration is reduced in the presence of inflammatory cytokines like such as necrosis factor-*α* and interleukin-1*β* [62, 82, 93, 94]. These cytokines induce an upregulation of the cell-cell adhesion molecule E-selectin [82]. The subsequent adhesion of tumor cells expressing the E-selectin counter-receptor sialyl Lewis x (CD15s) to endothelial E-selectin leads to an upregulation of stress-activated protein kinase-2 (SAPK2/p38) in endothelial cells [82] and triggers actin polymerization and actin reorganization into stress fibers [83] (Figure 4).

Chemokines and their receptors have an impact on leukocyte trafficking [95, 96] and tumor cell invasion [97]. The superfamily of chemokines consists of small cytokine-like proteins that induce cytoskeletal rearrangements in endothelial cells and leukocytes, the firm adhesion of leukocytes to endothelial cells, and the directional migration (chemotaxis) of leukocytes [95]. The involvement of chemokines in tumor-endothelial interactions and their effect on tumor cell mechanical properties during invasion are considerably less well understood.

We recently found that the expression of the chemokine receptor CXCR2 enhanced tumor cell motility in 3D extracellular collagen matrix. The expression of the CXCR2 influences other mechanical properties of the receptor bearing cells [6]. High CXCR2 expressing subcell lines derived from the human breast carcinoma cells MDA-MB-231 produced enhanced traction forces on fibronectin-coated 2D substrates [6]. CSK-dynamics, cell stiffness, and CSK-fluidity are enhanced [6].

**Figure 6:**
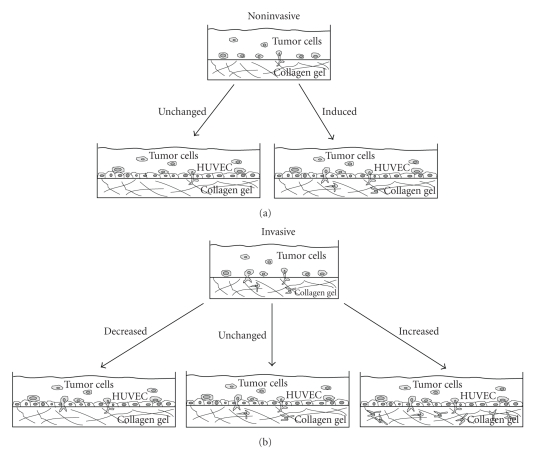
New role of the endothelium in the process of tumor cell invasion. (a) A human endothelial cell monolayer (e.g., HUVEC) cultured on top of a collagen fiber network-induced tumor cell invasion of cocultured human tumor cell lines. (b) The endothelium decreased the invasion of several invasive tumor cell lines (barrier for cell invasion), did not alter the invasion of tumor cells, or even enhanced tumor cell invasion into a collagen gel fiber matrix (enhancer for cell invasion).

Another impact of the CXCR2 receptor in the metastasis formation process could be the interaction with endothelial cells producing the CXCR2 ligands Gro-*β* and IL-8. Both chemokines enhance the transmigration and the followed invasiveness in vitro 3-D collagen fiber matrix assays [6]. Recently, it has been reported that Gro-*β* and IL-8 receptor (CXCR2) expressions on tumor cells are the key mediators responsible for the breakdown of the endothelial barrier function by enhancing tumor cell force generation and cytoskeletal remodeling dynamics (Figure 6). These results have to be expanded on other tumor cell types and further improved by using animal metastases models. All these different stimulations of the cells might underlie a common change of the cell's mechanical properties.

## 4. Role of Other Cell Types during Metastasis Formation

Several recent reports discussed the importance of platelets in adhering to single tumor cells and promote or induce their transendothelial migration [98–100], but in contrast to them, many other reports show clearly that single tumor cells are by themselves able to transmigrate through the endothelium [6–8, 62]. The platelets might be able to decorate single tumor cells and enhance their transmigration and invasion capabilities. But the question of do other cell types promote or induce tumor cell invasion and metastasis formation is still not answered fully. Do platelets guide tumor cells through the endothelium? For example, the role of mast cells either in solid tumors or in close neighborhood to tumors is poorly investigated. Did mast cells also promote or induce tumor cell transmigration or invasion? Do mast cells stimulate tumor cells to contract after histamine release? It has been reported that mast cells survive in close contact to endothelial cells and even proliferate there [101]. This might explain the increased numbers of mast cells in vascularized solid tumors [102–104]. Of course other cell types like eosinophilic granulocytes, lymphocytes, or monocytes/macrophages might also influence the mechanical properties of tumor cells either by secretion of contractile force stimulating (enhancing) or by relaxing the substances. The role of mast cells in tumor metastasis formation has to be ruled out by using biophysical tools as well as dual-color imaging animal metastasis models.

The presence of macrophages in a tumor has been correlated with a poor prognosis, but how macrophages are involved in hematogenous metastasis was unclear. Do macrophages change the mechanical properties of the tumor cells either by direct binding or by secretion of cytokines or by growth factors? Direct visualization of macrophages that assisted tumor cell intravasation in hematogenous metastasis of mammary tumors has been reported by using multiphoton microscopy [105]. Macrophages supported tumor cell motility toward blood vessels and tumor cell intravasation [105]. How this affects the mechanical properties of the tumor cells and their ability to generate contractile forces to move forward remain unclear and should be further investigated using biophysical tools, for example, traction microscopy. The dual-color fluorescence imaging technology distinguishes injected and fluorescently labeled tumor cells from the host stroma cells of transgenic mice and enables further insights in tumor-host interactions [106, 107].

## 5. Contractile Forces and the Role of Vinculin

### 5.1. Measurement of Contractile Forces

To move forward in a 3D matrix, the invading tumor cells need to generate contractile forces to overcome the viscous drag. Despite the composition and the enzymatic digestion of the matrix, the coordinated regulation of cell adhesion (adhesion strength) and deadhesion (including cytoskeletal remodeling) together with the generation of contractile forces determine the invasion speed in a 3D matrix. Our knowledge of cell migration, mechanical tensions, and forces is derived from studies of cells cultured on planar substrates (e.g., glass or polyacrylamide hydrogels). Previous reports showed how to measure traction forces during cell migration in 2D culture systems [108, 109] and were further improved to quantitative tools [110–113]. All the methods depend on the measurement of the deformations of an elastic substrate with known elastic modulus on which the cells adhered and spread. During this cell-matrix contact, the cell generates tractions and deforms the substrate. The tractions were computed from the substrate displacements visualized by embedded markers (fluorescent 0.5 *μ*m beads) using continuum mechanics theory. Measurement of the displacement field is accomplished by tracking these beads that are near the surface of the substrate. The spatial resolution of the traction map obtained with traction microscopy approaches is 1 *μ*m, which is sufficient to resolve the forces from individual focal adhesions [112]. Traction microscopy has brought new insights into tumor cell migration in particular [6, 86, 113]. The cells feel and respond to the stiffness of the extracellular matrix through dynamic regulation of integrin clustering, altered focal adhesion formation, and remodeling of the cell's cytoskeleton [40]. To conclude, contractile force generation and cell invasion are strongly influenced by the mechanical properties of the extracellular matrix [109].

### 5.2. Cell-Matrix Adhesions Protein Vinculin

For the generation of contractile forces, a cell needs to adhere to the 3D matrix via cell-matrix adhesions. The focal adhesion protein vinculin has been described to be structurally important connecting the extracellular matrix via cell-matrix adhesions and adjacent cells via cell-cell adhesions with the actin cytoskeleton by providing an intracellular linkage between the integrin receptor and the actin cytoskeleton via the focal adhesion formation [114–117]. The vinculin protein is encoded by 1066 amino acids, has a molecular weight of ~117 kDa, and binds to many other focal adhesion and cytoskeletal proteins including talin, paxillin, tensin, zyxin, VASP, *α*-actinin and actin [118].

The function of vinculin has been largely analyzed in cells cultured in 2D systems. Vinculin knock out (k.o.) mouse embryonic fibroblasts (MEFs) as well as vinculin k.o. mouse embryonic carcinoma cells (F9 cells) exhibit a more round cell shape, show fewer and less stable lamellipodia, but similar filopodia compared to vinculin-expressing MEFs (wild type) [116, 119, 120]. Focal adhesion complexes of vin-/- cells are smaller and consist of increased tyrosine-phosphorylated proteins like talin, *α*-actinin, FAK, and paxillin without increasing total protein content [116, 121]. Vinculin-deficient fibroblasts (MEFvin-/-) and carcinoma cells (vin-/-) show reduced adhesion to extracellular matrix proteins like fibronectin, vitronectin, laminin, and collagen as well as a two-fold increase in motility and three-fold increase in focal adhesion kinase activity compared to wild-type cells [121, 122]. All the described studies were performed in 2D migration or 2D adhesion assays and did not properly mirror the in vivo situation of cells invading and contracting the 3D tissue environment during the malignant progression of tumor disease involving tumor outgrowth and metastasis formation.

Vinculin's function in tumor disease has so far hardly been investigated. We recently reported that vinculin functions as a mechano-coupling protein, and that its tail-domain acts as a regulator for contractile force generation [86].

In a recent study, we examined whether vinculin is involved in cell invasion into a 3D extracellular matrix [123]. We found that vinculin enhanced the generation of tractions, and that this mechanical parameter despite all other mechanical parameters (e.g., cell adhesion, detachment, and matrix degradation) plays a prominent role in cell invasion in a 3D matrix to overcome the steric hindrances, but not on a 2D substrate, where the viscous drag is negligible. Future analyzes of the transmission of forces in cells via focal adhesions might help to determine the prerequisites of the invasive potential of a tumor cell.

## 6. How Can Mechanical Parameters of the Cells Help to Improve Tumor Diagnosis?

Several reports describe a relationship between tumor cell mechanical properties and their malignant potential in promoting metastasis formation [6, 43, 124]. The measurement of only a single cell mechanical parameter might not be sufficient to predict the invasiveness of tumor cells nor their metastasis formation potential. Many factors may be involved in promoting tumor cell invasion (Figure 2) and tumor cell transendothelial formation (Figure 5). The mechanical properties of the tumor cells as well as their adhesion receptor, sheddases, or enzyme expressions together determine the ability to invade a tissue and to transmigrate the endothelium (Figures 2 and 5). Only small fraction of tumor cell lines is able to invade a 3D extracellular matrix [6]. To provide a proper analysis tool to determine the invasive or metastasis potential of a tumor, it is first important to isolate subcell fractions of isolated primary tumor cells using well-defined mechanical parameters (contractile force generation). These obtained primary tumor cells can be analyzed for their metastatic potential by using metastatic animal models.

## 7. Role of Clinically Relevant Metastatic Animal Models

Orthotropic metastatic animal models can be used to discover anticancer drugs and to determine drugs' efficiency [71]. These models are established for a wide variety of tumors including spontaneous metastatic bone models of prostate cancer, breast cancer, and lung cancer as well as spontaneous liver and lymph node metastastic models of colon, pancreatic, stomach, ovarian, bladder, and kidney cancer [71]. These models present a new generation of rodent tumor models and differ from transgenic tumor models or subcuntanenously-growing human tumors in immunodeficient mice, because the latter models did not represent sufficient models in regard to metastasis formation or drug sensitivity [71]. The advantage of novel orthotropic metastatic models is that histologically intact fragments of human cancer—directly taken from the patient—were implanted to the corresponding organ of immunodeficient mice. This allows researchers to determine tumor growth and metastatic potential of transplanted tumors [125, 126]. These orthotropic metastatic animal models can be combined with measurements of the cells mechanical properties and the mechanical properties of the transplanted tissue. To understand the diverse roles of the endothelium in the tumor metastasis, a transgenic nude mouse model visualizing tumor angiogenesis could be helpful. It is based on the nestin-promotor-linked green fluorescent protein because many of the newly formed blood vessels originate from hair-follicle stem cells that express nestin. The transcription of GFP indicates newly formed blood vessels in the animal [127].

## 8. Conclusions and Future Directions

In summary, this paper presented a new function of the endothelium in inducing or enhancing tumor cell invasion. It discussed the two described pathways for tumor cell metastasis formation and pointed out the importance of cell-matrix and cell-cell adhesion receptors as well as chemokine receptors for tumor-endothelial cell interactions. It focused the view on the mechanical properties of tumor cells as well as that of endothelial cells during the process of metastasis formation. It discussed in vivo animal models to visualize vascular and lymphoid metastases formation of single injected tumor cells or implanted orthotropic tissue resections.

The main new direction of tumor cell invasion and metastasis research is to measure the ability of tumor cells to generate contractile forces in a 3D environment and to determine the malignancy of a tumor using in vivo animal models. The mechanical measurements of tumor cell subpopulations in patient's liquids or tissue samples might help to determine whether a tumor is able to form metastases. A future direction of tumor metastasis research is to focus on the diverse roles of the endothelium. The combination of biophysical tools for mechanical properties analysis of tumor and endothelial cells, the generation of mechanical diverse tumor cells, and with their injection into nude mice using the dual-color in vivo imaging method will be extremely helpful in developing new diagnosis tools and drugs reducing tumor metastases.
